# Social safeness and eating disorder symptoms: a correlational study exploring associations of social safeness, eating disorder symptoms, social support and shame in a non-clinical young adult sample

**DOI:** 10.1186/s40337-024-01057-1

**Published:** 2024-07-22

**Authors:** Jasmin Langdon-Daly, Hannah Chuang, Anna Marie Handke

**Affiliations:** Department of Psychology, University of Bath, Claverton Down, Bath, BA2 7AY UK

**Keywords:** Eating disorders, Eating disorder symptoms, Social safeness, Social support

## Abstract

**Background:**

A greater experience of “social safeness” in social relationships has been associated with reduced general psychopathology. This association appears to be independent of the perceived level of actual social support. The tripartite model of emotion suggests that experiences of social safeness may be associated with increased activation of the ‘soothing system’, inhibiting the experience of threat and shame. Associations of eating disorder (ED) pathology and shame have been well established. This study aims to answer the questions: Is there an association with experience of social safeness and ED symptoms in a non-clinical sample? Are any associations independent of perceived or received social support?

**Methods:**

A non-clinical sample of 80 young adults (aged 18–25) completed an online survey. The survey included measures of ED symptoms, social safeness, perceived and received social support and shame. Correlation and hierarchical regression analyses were conducted to explore cross-sectional associations between variables.

**Results:**

Increased sense of social safeness was strongly correlated with reduced ED symptoms. Social safeness explained unique variance in ED symptoms independent of received and perceived social support. Shame and ED symptoms were positively correlated, while shame and safeness were negatively correlated.

**Conclusions:**

This study presents evidence of an association between the experience of social safeness and ED symptoms. The impact of the emotional experience of social safeness appears independent of current social support, and may be associated with increased activation of the soothing system, and reduced activation of the threat system and experiences of shame, as in the tripartite model of EDs. Further research could explore these associations in clinical populations and explore whether reduced social safeness is a risk factor for the development / maintenance of EDs, or could be a useful target for ED interventions.

**Plain english summary:**

This study looked for a possible link between having a greater general experience of ‘safeness’ in social relationships, and reduced eating disorder symptoms, in a healthy young adult sample. Eighty young adult participants completed online questionnaire measures of eating disorder symptoms, social safeness, social support, and shame. Having a greater sense of ‘social safeness’ was strongly correlated with having lower eating disorder symptoms. This effect seemed to exist independently of the level of social support someone reported. Shame was positively correlated with ED symptoms and negatively correlated with social safeness. These findings are consistent with the ‘tripartite model’ of emotion: the emotional experience of social safeness may be linked with increased activation of the soothing system and reduced activation of shame. A link between reduced experience of ‘social safeness’ and eating disorder symptoms may have useful implications for understanding and intervening with eating disorders.

## Background

The role of healthy interpersonal contexts and relationships in preventing psychopathology is widely established in the clinical and psychosocial literature [1–3]. According to Gilbert et al.’s tripartite model [3], fundamental needs of human survival, such as the need to feel socially accepted and valued, are served through the interaction of three systems of affect regulation: (1) The threat-protection system detects and responds to potential cues of danger, e.g., threats to one’s sense of belonging, (2) the resource-seeking system enables the seeking out of desirable resources needed for survival, e.g., alliances and companionship, and (3) the contentment-soothing system enables the regulation of emotional safeness and calmness [4]. The third system plays a crucial role in enabling healthy socialisation, feelings of interpersonal connection, and stimulates necessary social behaviour for forming close relationships [5].

Gilbert et al. [3, 6, 7] characterises a form of positive affect arising from the action of this third system as “social safeness”—an individual’s feelings of connectedness, warmth, and feeling cared for within their social world. Informed by attachment theory [8], Kelly and Dupasquier [9] suggest that feelings of warmth and connection experienced in childhood significantly influence the development of the contentment-soothing system. Neuroscience research finds that primary relationships nurtured from birth function as critical regulators of positive affect systems and produce a profound sense of comfort similar to social safeness [10].

It has been suggested that experiences of social safeness nourish healthy emotional states needed to manage adversity and defend against psychopathology [7, 10]. A number of studies have explored associations between experiences of social safeness and forms of psychological distress and psychopathology. Reduced self-reported social safeness has been correlated with reduced self-esteem, and increased self-criticism, attachment disturbance, and self-report of depressive and personality disorder symptoms [2]. These associations were found to be stronger than correlations with measures of simple positive or negative affect.

While few studies have explored links between social safeness and eating disorders (ED), two papers report that higher levels of social safeness were significantly correlated with reduced ED symptoms in a non-clinical sample of Portuguese women [4, 11]. Models of ED pathology suggest that threat-protection and resource-seeking systems dominate in EDs, leaving the contentment-soothing system underutilised [5, 12]. This dominance is exhibited in individuals with EDs in their primarily negative emotional states and highly driven attitudes towards achieving thinness, abiding by eating rules, and immense satisfaction in their restrictive accomplishments [13–15].

Literature also frequently associates a lack of social support with psychopathology. Social support is defined as a social network’s provision of emotional and psychological resources intended to protect individuals’ health in times of stress [16]. Stress buffering theory implies that support from members of one’s immediate social network acts as a protective buffer against the psychological effects of stress on mental health [17, 18]. Theorists distinguish between two major types of social support: Received social support refers more to the quantity of supportive behaviours provided, whilst perceived social support refers to the perception of the general availability and overall satisfaction of support [19].

Links between social support and ED symptoms do not appear to be straightforward [20]. For example, qualitative findings from the perspectives of 22 recovered women showed that social support, regardless of the amount, was only helpful if supporters emitted a stable and non-judgemental presence that fuelled feelings of connectedness. Social support that made them feel misunderstood or focused too heavily on recovery was perceived as harmful and threatening [21]. Several studies demonstrate the overall tendency of individuals with EDs to feel dissatisfied with and seek out less social support [22]. This raises the question of whether the positive effect of social support requires feelings of social safeness.

Gilbert et al. [3] imply that social safeness still has significant protective qualities even in the context of low social support. Whilst social support is considered a behavioural / cognitive construct that based on beliefs on whether individuals feel supported and receive support when needed, social “safeness” is set apart as a unique, deep-rooted, emotional experience related to interpersonal warmth and calmness [2].

Gilbert [23] theorises that individuals with low social safeness experience significant fears of judgement and trusting compassion in other people and, therefore, tend to discern less social support [9]. Social support may operate in a way that is subservient to or a by-product of the feeling of social safeness. Leonidas and Santos [20] suggest that internal states of social insecurity specific to people with EDs cause them to interpret support as unfavourable or triggering, and therefore seek out less support. Initial healthy levels of social safeness may be needed to make one feel less threatened and more inclined to receive and benefit from social support [9].

Shame is a powerful self-conscious and negative emotion associated with social rejection and a sense of failure [3, 12], which has consistently been linked to a range of psychopathologies including EDs [12, 24]. Clinical ED samples consistently score higher on measures of shame than non-clinical samples, with scores being positively associated with the severity of symptoms. It has been suggested that any associations between social safeness and ED symptoms may act fully or in part through the impact of social safeness on shame [4]. Higher levels of shame have been associated with greater difficulties maintaining interpersonal relationships and feeling less socially safe [23]. In the tripartite model of emotion, shame is described as a threat-based emotion, linked to the threat of social rejection [25]. The experience of shame is therefore described as subject to regulation by the action of the connect/soothe system, and potentially by the experience of social safeness. It is plausible that interacting patterns of reduced social safeness, increased shame, and withdrawal from social support, could play a role in the development and maintenance of EDs.

### Study aims and hypotheses

The current study aims to investigate the relationship between social safeness and EDs, to answer the questions:

1. Is there an association of experience of social safeness and ED symptoms in a non-clinical sample?
2. Are any associations independent of perceived or received social support?

The present study tested the following hypotheses:

1. Social safeness will be significantly negatively correlated with ED symptoms.
2. Received and perceived social support will be significantly negatively correlated with ED symptoms.
3. Social safeness will account for a significant proportion of variance in ED symptoms, independent of received and perceived social support.
4. Shame will be significantly positively correlated with ED symptoms and negatively correlated with social safeness.

## Methods

### Design

The study used a cross-sectional correlational design to explore associations between self-report measures of ED symptoms, social safeness, social support and shame.

### Participants

Participants were 80 young adults aged between 18 and 25, including 55 females, 17 males and 8 people with other gender identities. The mean age of participants was 21.78 years (SD = 1.91 years). Inclusion criteria were anyone capable of providing informed consent in this age range, as it reflects the average onset of EDs and is widely known as a period of high risk of ED development [26]. The self-reported ethnic background of participants was 16.2% Asian/Pacific Islander (*n* = 13), 72% White (*n* = 57), 1.2% Hispanic (*n* = 1), 7.5% Multiple ethnicities/Other (*n* = 6), and 3.7% (*n* = 3) preferred not to say.

Participants were recruited using social media to yield a general population sample. In order to include within the sample some participants who reported higher levels of ED symptoms, social media advertisements were posted both broadly and also directly to some public Twitter user accounts listing Edtwt in their biography —An abbreviation for “Eating Disorder Twitter,” referring to a Twitter community for users struggling with ED or ED symptoms.

### Measures

#### Eating disorder examination questionnaire (EDEQ)

The EDEQ is a 28-item measure that assesses the frequency of ED symptoms over the past 28 days [27]. It assesses overall ED symptom severity through a global score of 0 to 6, with higher scores expressing greater symptom severity. The EDEQ calculates global scores by finding the mean of scores on four subscales: Restraint, Eating Concern, Weight Concern, and Shape Concern. Participants are asked to answer all questions in reference to the past 28 days. The EDEQ demonstrates adequate internal consistency for the subscales and global score, with Cronbach’s alphas ranging from 0.70 to 0.93 [28]. In the present study, the Cronbach’s alpha value was 0.98. A clinical cut-off of 2.8 has been suggested for this measure [29].

#### Social safeness and pleasure scale (SSPS)

The SSPS is an 11-item measure developed to assess the degree to which individuals experience social safeness, defined as feelings of warmth, connectedness, acceptance, and being cared for in their social world [6]. Participants report their agreement regarding 11 statements, e.g., “I feel a sense of belonging.”, on a 5-point Likert scale from 1 (almost never) to 5 (almost all the time). The SSPS demonstrates adequate internal consistency with a Cronbach’s alpha coefficient of 0.91. In the present study, the Cronbach’s alpha value was 0.89. The items “I feel secure and wanted,” “I feel a sense of belonging,” and “I feel accepted by people” demonstrate the highest internal consistency [2], implying that the SSPS highly encapsulates the dimensions of social safeness, e.g., warmth and acceptance.

#### Social provisions scale (SPS)

Perceived and received social support was assessed using Kelly et al.’s [2] amended 6-item version of the SPS [30]. Participants are asked to rate their responses on a 4-point Likert scale from 1 (strongly disagree) to 4 (strongly agree). Perceived and received social support are each assessed by three items: Guidance (i.e., “There is a trustworthy person I could have turned to for advice or guidance if I were having problems today,” and “To what extent did another person(s) provide you with advice or guidance today”), Reliable Alliance (i.e., “There are people I could have counted on to come to my assistance if I really needed it today,” and “To what extent did another person(s) come to your assistance today”), and Attachment (i.e., “I have close relationships that could have provided me with a sense of emotional security and well-being if I were upset today,” and “To what extent did another person(s) provide you with a sense of emotional security and well-being today”). The original SPS demonstrates high reliability with a Cronbach’s alpha of 0.82 to 0.93 [31], and the amended 6-item version demonstrates medium reliability with a Cronbach’s alpha of 0.85 for PSS and 0.61 for RSS [2]. In this paper, the Cronbach’s alpha values were 0.83 for PSS and 0.86 for RSS.

#### Other as Shamer scale (OAS)

The OAS [32] is a measure of external shame, which has been used in the literature linking shame and EDs [4]. The measure contains 18 items scored on a 4-point Likert scale (0 = never, 4 = almost always) with items such as “People see me as unimportant compared to others”. The original study demonstrated a Cronbach’s alpha of 0.92 and a mean score of 20 (SD = 10.1) in a non-clinical sample. Further studies have confirmed good validity and reliability [33]. In the present study, the Cronbach’s alpha value was 0.96.

### Procedure

Data were collected via an online cross-sectional questionnaire using a convenience sampling method between 6 June 2023 and 26 July 2023. A recruitment advertisement for the study with a link to an online questionnaire adapted on Qualtrics [34] were posted and distributed via social media and chatting apps, including WhatsApp, Instagram, and Twitter. Participants were incentivised with the option to enter their email to take part in a prize draw for the chance to win a £20 voucher after taking part in the study.

After reading participant information and indicating consent to participate, participants completed an online survey consisting of the EDEQ, SSPS, SPS and OAS, a demographics questionnaire that asked for age, gender, and ethnicity, as well as entering contact details for the prize draw. Typical time required to complete the study was about 10–15 min, although some participants took significantly longer than this (mean = 16.11 min, median = 9.85 min). A debrief sheet with supportive resources, including contacts of mental health and eating disorder organisations and services, followed the end of the study.

### Statistical analysis

A priori power analysis was conducted using G*Power version 3.1 [35] for a hierarchical multiple regression with 3 predictor variables. To achieve 80% power for detecting a medium effect size (*f* = 0.15, based on medium to large effect sizes found in Kelly et al. [2, 36]) at a significance level of *a* = 0.05, the suggested sample size was *N* = 77.

All statistical analyses were calculated using IBM SPSS Statistics [37]. Only data from participants who completed all study measures in full were included. One participant completed nearly all measures but did not complete the SPS and so was excluded from the study sample, leaving a final sample size of 80.

Pearson’s correlations were calculated to determine the magnitude and significance of the relationship between ED symptoms (Global EDEQ score), social safeness (SSPS scores), received and perceived social support (SPS scores), and shame (OAS).

A hierarchical multiple regression was conducted to assess whether social safeness explained unique variance in ED symptoms controlling for both types of social support. Received and perceived social support were entered as control variables in Step 1 and social safeness was entered in Step 2.

## Results

### Descriptives

Eighty participants completed all study measures and were included in the study. Mean and standard deviation for scores on the EDEQ, SSPS, SPS and OAS are presented in Table 1. Forty-three participants (54%) were presented with zero to mild ED symptoms, and 37 participants (46%) presented with clinically significant ED symptoms within the past 28 days (above EDEQ cut-off score = 2.8 [29]).

**Table 1:** Means and standard deviations of scores on all measures

Variable	Mean	SD
1. Eating Disorder Symptoms (EDEQ)	2.67	1.98
2. Social Safeness (SSPS)	24.31	10.80
3. Perceived Social Support (SPS)	9.67	2.35
4. Received Social Support (SPS)	7.87	2.64
5. Shame (OAS)	30.95	20.36

*Note* *N=80*

### Assumptions tests

**Fig. 1:**
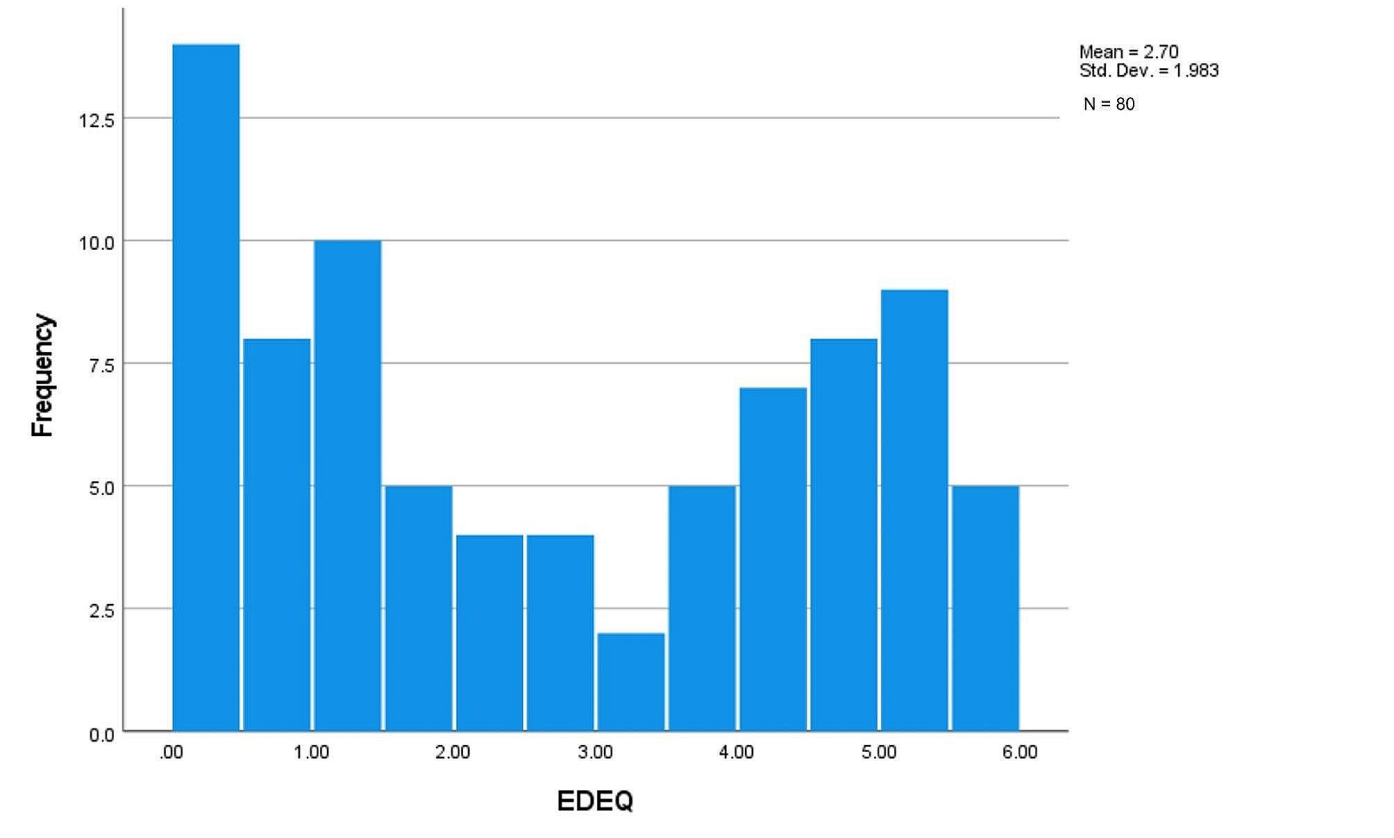
Histogram showing distribution of EDEQ scores in this sample. Note: Cut-off for clinically-significant symptoms = 2.8 [29]

An analysis of standard residuals identified zero outliers (Std. Residual Min = -2.25, Std. Residual Max = 2.27). Collinearity tests indicated no multicollinearity violations (Minimum Tolerance = 0.38; Maximum VIF = 2.56) [38]. The data also met the assumption of independent errors (Durbin-Watson value = 1.43) and a histogram showcasing standardised residuals indicated that errors were reasonably normally distributed. ED symptoms (EDEQ) were significantly non-normal *W*(80) = 0.89, *p* < .001 according to the Shapiro-Wilk test (see Fig. 1) and a scatterplot of standardised residuals revealed no violations of homogeneity of variance and linearity assumptions. The assumption of non-zero variances was also met (Minimum Variance = 3.89).

#### Hypothesis 1, 2 and 4

***ED symptoms will be negatively correlated with social safeness, received and perceived social support, and positively correlated with shame***.

**Fig. 2:**
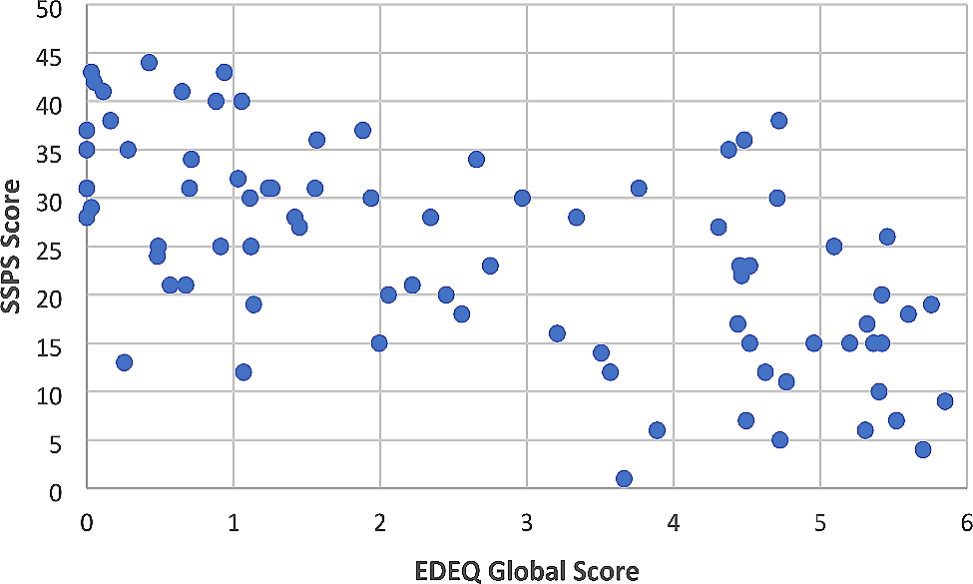
Association between eating disorder symptoms and social safeness

Pearson’s correlations were calculated to assess the linear relationships between all variables. Results of these analyses are reported in Table 2. Results indicated that ED symptoms were significantly negatively correlated with social safeness (*r*=-.61, *p* < .001), see Fig. 2. ED symptoms were significantly negatively correlated with perceived and received social support, and significantly positively correlated with shame. Shame was significantly negatively correlated with social safeness.

**Table 2:** Correlations of study variables

Variables	1.	2.	3.	4.	5.
1. Eating Disorder Symptoms (EDEQ)	--				
2. Social Safeness (SSPS)	− 0.61***	--			
3. Perceived Social Support (SPS)	− 0.45***	0.70***	--		
4. Received Social Support (SPS)	− 0.44***	0.66***	0.72***	--	
5. Shame (OAS)	0.72***	− 0.73***	--	--	--

*Note* A. *N=80*

B. Statistical significance: ***p < .001 (1-tailed)

#### Hypothesis 3

***Social safeness accounts for a significant amount of variance independent of received and perceived social support***.

Hierarchical multiple regression results are summarised in Table 3. In the first step of the regression, received and perceived social support were added. The results of the first step revealed a statistically significant model *F* (2, 77) = 11.77; *p* < .001. Received and perceived social support when combined accounted for 23% of the variation in ED symptoms.

**Table 3:** Hierarchical multiple regression analyses Predicting ED symptoms (EDEQ)

	*R*	*R*₂	*R*₂ change	b	SE	β	t
Model 1 Perceived Social Support Received Social Support	0.48	0.234***		− 0.23 − 0.17	0.12 0.10	− 0.28 − 0.23	-1.95 -1.65
Model 2 Perceived Social Support Received Social Support Social Safeness	0.61	0.372***	0.138***	− 0.02 − 0.04 − 0.10	0.12 0.10 0.02	− 0.02 − 0.06 − 0.54***	− 0.18 − 0.43 -4.0

*Note*  A. Statistical significance: *p < .05; **p < .01; ***p < .001

*B. Step 1 predictors: (Constant), Received Social Support, Perceived Social Support*

*C. Step 2 predictors: (Constant), Received Social Support, Perceived Social Support, Social Safeness*

For the second step, social safeness was added to the analysis. The results of the second step revealed that the overall model—received social support, perceived social support, and social safeness as predictors, was significant (*F* (3, 76) = 15.01; *p* < .001). The combination of the three predictors explained 37% of the variance in ED symptoms. Social safeness accounted for a significant and unique amount (13%) of the variance in ED symptoms controlling for received and perceived social support (*R₂* change = 0.13; *F* (1, 76) = 16.68; *p* < .001).

On an individual level, the regression coefficient associated with social safeness *β =* − 0.54, *p* < .001 suggests that social safeness accounted for a significant amount of unique variance in ED symptoms, in which every additional unit of social safeness led to an approximate decrease of 0.54 units in ED symptoms controlling for received and perceived social support. Received and perceived social support when investigated separately, did not yield statistically significant Beta values, (*β =* − 0.06, *p* > .05 and *β =* − 0.02, *p* > .05 respectively).

Social safeness was the only predictor variable that independently explained variance in ED symptoms (13%). Received social support and perceived social support only explained variance when taken together (23%) or with social safeness (37%). Hypothesis 3, that social safeness would account for a significant amount of variance independent of received and perceived social support was accepted.

## Discussion

This study aimed to explore relationships between social safeness, social support, shame and ED symptoms. The findings supported the proposed hypotheses. ED symptoms were significantly negatively correlated with social safeness. Lower levels of social safeness were associated with higher levels of ED symptoms. ED symptoms were negatively correlated with social support, although less so than with social safeness. Hierarchical regression indicated that social safeness explained a significant proportion of the variance independently of social support, suggesting an independent effect. ED symptoms were positively correlated with shame. Increased levels of shame were associated with increased ED symptoms, and lower levels of social safeness.

The association of lower social safeness with higher ED symptoms is consistent with findings of Pinto et al. [4, 11], and with research linking lower social safeness with other forms of psychopathology [2]. These findings fit with the tripartite model of emotion [3], and with the tripartite model of EDs which suggests an underactive content/ soothing system in EDs, combined with overdominance of the threat and drive systems [12]. Increased experience of anxiety and threat is well-documented in people experiencing EDs, and considered to be important to the development and maintenance of EDs [13]. Experiences of care from others in developmental history and in current relationships, associated with an experience of social safeness, are thought to be linked to increased activation of the connect/soothe system, which may inhibit threat (and drive). Where the experience of social safeness is reduced, this inhibition could also be reduced, leading to increased threat/ drive activation and driving ED symptoms.

While previous research has highlighted a protective effect of social support against EDs [20], the effect of social safeness here was significant independently of current reported levels of social support. This finding can be considered in the context of research which suggests that for some with EDs, social contact and support may be experienced as aversive or unwanted, or not having a protective effect [21, 22]. It is consistent with an account of social safeness as an emotional interpersonal experience which has its roots in the attachment and relational history of an individual, rather than just reflecting the nature of their current relationships [2, 3, 9]. It is plausible that with a reduced sense of social safeness, an individual with an ED may be less able to seek out, perceive and benefit from the protective effect of social support [20]. Conversely, it is possible that developing and supporting someone’s emotional experience of social safeness may exert a positive effect regardless of the current level of actual social support available to them [3].

The strong correlation between increased shame and ED symptoms is consistent with the existing literature [12, 24]. Social safeness is strongly negatively correlated with shame. The findings of this research are consistent with those of Ferreira et al. [4], suggesting a possible role for reduced social safeness in this experience of increased shame. This is again consistent with the tripartite model, which conceptualises shame as an emotion grounded in the action of the threat system [3]. Where there is reduced social safeness, there may be reduced inhibition of the threat system from the soothing system, allowing for increased shame, and potentially therefore increased ED symptoms.

These ideas may have important implications for models of ED development. They could also suggest strengthening an individual’s emotional experience of social safeness as a helpful target for intervention in psychological treatments for EDs [7]. This could be done through exercises to develop the individuals emotional experience of safeness (such as compassion exercises), through work exploring an individual’s current relationships and relational history, or through directly using the therapeutic relationship to create a relational experience of consistent, safe support. This may be particularly beneficial where an individual is reporting high levels of shame.

A major limitation of this study is the cross-sectional and correlational nature of the associations explored. This severely curtails the ability to draw conclusions related to causality from the findings. As such, any conclusions should be considered as preliminary evidence of a potential association, suggesting avenues for potential longitudinal studies. The measures used in the study also refer a range of timescales – in particular the SPS assesses very current (today) support, while the SSPS refers to a broader or non-specific timescale. Studies repeating these measures daily have found similar levels of moderate within-participant reliability for these measures (*r* = .66 and *r* = .77 respectively) [2], suggesting that responses to both measures fluctuate over time while showing some consistency within individuals, suggesting this should not be considered a major limitation to the interpretation of these findings. The population studied was not a clinical sample, although individuals in the study did report ED symptoms consistent with the ‘clinical range’ on the EDEQ. The sample was also completely self-selected, and the use of social media in recruitment means the population is likely to be likely to be representative of the social networks of the researchers (e.g. possibly high numbers of students and individuals with high education levels).

Further research could address the limitations of this study by looking for longitudinal associations, using a larger and less self-selected sample to be more representative of the general public, or using a clinical sample. If the associations here are found to be replicable, studies could explore factors which influence the development of a stronger or weaker sense of social safeness, and the role of these factors in the development of ED. Clinical research could investigate the nature of social safeness in the therapeutic relationship, the impact of social safeness on therapeutic outcome, or conversely the impact of therapeutic actions on the experience of social safeness.

## Conclusions

The results of this cross-sectional study indicate that in a non-clinical, young adult sample, an experience of greater social safeness is associated with lower reported levels of ED symptoms. This association appears to be independent of current levels of reported social support. Increased shame was correlated with increased ED symptoms and reduced social safeness. The results of this study may have implications for models of ED risk and maintenance. The emotional experience of reduced social safeness may influence the experience of social support in individuals with EDs, and be associated with reduced activation of the soothing system, and increased experience of shame and activation of the threat system, as in the tripartite model of EDs. If replicated longitudinally, these findings could be of interest in the development and refinement of interventions for EDs.

## Data Availability

The datasets used and/or analysed during the current study are available from the corresponding author on reasonable request.

## Abbreviations

ED: Eating disorder
EDEQ: Eating Disorder Examination Questionnaire
OAS: Other as Shamer Scale
SPS: Social Provisions Scale
SSPS: Social Safeness and Pleasure Scale
